# Alterations in the preferred direction of individual arm muscle activation after stroke

**DOI:** 10.3389/fneur.2023.1280276

**Published:** 2023-09-22

**Authors:** Yoon No G. Hong, Jinsook Roh

**Affiliations:** Department of Biomedical Engineering, University of Houston, Houston, TX, United States

**Keywords:** preferred direction, muscle synergy, motor impairment, qualities of motor performance, stroke, isometric, coordination

## Abstract

**Introduction:**

Stroke survivors have challenges appropriately coordinating the multiple muscles, resulting in a deficit in motor control. Therefore, comprehending the mechanism underlying abnormal intermuscular coordination becomes crucial in developing effective rehabilitation strategies. Quantitative analyses have been employed at pairwise or multi-dimensional levels to understand the underlying mechanism of abnormal intermuscular coordination and its relationship to motor impairment. However, how alterations in individual muscle activation contribute to abnormal intermuscular coordination, motor impairment, and motor performance remains unclear. Thus, we investigated the alterations in the preferred direction of individual muscles after stroke and their relationship with stroke-induced changes in intermuscular coordination, clinical motor impairment, and qualities of motor performance during isometric force generation in the upper extremity.

**Methods:**

Twenty-four stroke survivors and six age-matched controls were recruited and performed isometric force target matches while recording electromyographic signals from eight upper limb muscles. We determined the preferred activation direction of each muscle, evaluated abnormal intermuscular coordination through a muscle synergy analysis, assessed motor impairment using upper extremity Fugl-Meyer Assessment scores, and examined motor performance characteristics defined by force trajectory features.

**Results:**

The post-stroke alterations in the preferred direction of the brachioradialis, anterior, middle, and posterior deltoid were correlated with the motor impairment level and attributed to the changes in muscle synergy characteristics. Only alterations in the preferred direction of the brachioradialis and posterior deltoid activation in forward-backward and upward-downward axes were associated with the qualities of isometric force generation, respectively.

**Discussion:**

These findings imply that alterations in the preferred direction of individual muscle activation contribute to various aspects of motor deficit following stroke. This insight may serve as a foundation for the development of innovative stroke neurorehabilitation approaches that take into account specific attributes of individual muscle activation, including their preferred activation direction.

## 1. Introduction

Stroke is a leading cause of long-term disability in the United States and worldwide, with a significant portion of stroke survivors experiencing chronic motor impairments in their upper extremities, significantly restricting their daily activities (1, 2). A prominent deficit in stroke survivors’ motor control arises from abnormal intermuscular coordination (3–6). Effective movement coordination is a vital skill that enables individuals to navigate the extensive degrees of freedom associated with motor redundancy (7) or abundance (8). Stroke survivors often have challenges executing complex motor tasks appropriately because of the impaired ability to activate and coordinate multiple muscles normally (9, 10). Therefore, comprehending the mechanism underlying abnormal intermuscular coordination becomes crucial in developing effective rehabilitation strategies that restore motor function and enhance the overall quality of life for stroke survivors.

Many studies have examined abnormal intermuscular coordination following stroke, aiming to unravel the underlying mechanisms contributing to motor impairments. For the upper extremity, abnormal intermuscular coordination was introduced and qualitatively described based on the visual observation of characteristic movement or postural patterns after stroke (11, 12). Stroke survivors often exhibit stereotypical movement patterns characterized by simultaneous shoulder abduction and elbow flexion (Flexion synergy) or shoulder adduction and elbow extension (Extension synergy). Later, quantitative analyses have been employed at pairwise or multi-dimensional levels to characterize intermuscular coordination and its relationship to motor impairment. For example, previous human studies have shown that increased co-contraction of antagonistic muscle pairs in a single joint, such as the wrist or elbow joint, correlates with the severity of motor impairment after stroke (13–16). Also, unique co-activation patterns between pairwise muscles in the elbow and shoulder joints have been identified in the paretic limb after stroke (3). Recent studies have utilized dimensionality reduction techniques to identify abnormal co-activation patterns, called abnormal muscle synergies, following stroke (5, 6, 17–19). They also examined an association between the abnormal muscle co-activation and other motor impairments, such as abnormal compensatory shoulder abduction and elevation during force target matches at the hand (5, 6), abnormal force coupling under isomeric conditions (17), and clinical motor impairment assessment scores (5, 6, 18, 19). However, to comprehensively understand alterations in intermuscular coordination after stroke, it is necessary to investigate how alterations in individual muscles contribute to abnormal co-contraction or co-activation post-stroke.

Investigating the preferred direction of muscles is a valuable approach to studying the alterations in individual muscle activation related to abnormal intermuscular coordination involving multiple muscles. The concept of a preferred direction has been initially utilized to explore the relationship between motor cortical activity and movement direction. Previous studies have reported that each single-cell activity in the motor cortical area exhibits a peak discharge rate in a distinct preferred direction (20–22). Similarly, previous studies have shown that muscle activity is directionally tuned (22–24). Stroke-induced damage would result in changes in the preferred direction of muscles. A previous stroke study demonstrated consistent and statistically significant shifts in the preferred direction of the paretic limb during isometric force generation compared to the contralateral limb (3). However, the precise relationship of these shifts to intermuscular coordination, motor impairment, and the qualities of motor behavior remains unclear.

Upper limb rehabilitation has been developed to target abnormal intermuscular coordination, specifically addressing co-contraction, co-activation, or muscle synergies. In the context of using surface electromyographic (EMG) signals for therapeutic intervention (e.g., myoelectric computer interface for stroke rehabilitation), a fundamental concept involves mapping the activation magnitude of individual muscles involved in abnormal coordination to the displacement of a cursor on display in different directions to decrease the abnormal co-activation (25, 26). Another intervention concept involves mapping sets of motor modules, also known as muscle synergies, to different directions of cursor movement on display. This strategy attempts to enhance the modulation of the activation of motor modules (27). The previous studies have successfully mapped the activation of individual muscles or motor modules to specific directional movements of the cursor to provide visual feedback on motor performance. However, they did not consider the importance of tuning individual muscle or motor module activation in the appropriate movement or force control direction. Understanding the relationship between the altered preferred direction of muscles and impaired intermuscular coordination after stroke is important to optimize motor neurorehabilitation approaches targeting abnormal intermuscular coordination for stroke survivors.

This study aimed to investigate the alterations in the preferred direction of individual muscles after stroke and their relationship with stroke-induced changes in intermuscular coordination, clinical motor impairment, and qualities of motor performance during isometric force generation in the upper extremity. We hypothesized that stroke survivors would exhibit alterations in the preferred direction of individual muscles, and these changes would be associated with abnormal intermuscular coordination as quantified by muscle synergies, motor impairment assessed by upper extremity Fugl-Meyer Assessment (FMA-UE) scores, and qualities of motor performance measured by force trajectory features. By exploring these associations, we aimed to understand better how alterations in the preferred direction of individual muscles contribute to the overall motor deficits observed following stroke.

## 2. Methods

### 2.1. Participants

Six age-matched neurologically intact participants and 24 chronic stroke survivors with mild to severe motor impairment (eight mildly impaired, FMA-UE > = 50; eight moderately impaired, 26 < = FMA-UE < 50; eight severely impaired, FMA-UE < 26; scores out of 66) were reanalyzed for the current study (5, 6). The demographics of the participants are described in Table 1. All participants signed an informed consent form approved by the Northwestern University Institutional Review Board.

**Table 1:** Participant demographics (mean ± std).

Group	Age	Months after stroke	FMA-UE (/66)	Sex (M/F)	Side affected (L/R)
Mild stroke	55.6 ± 9.5	51.0 ± 24.6	55.3 ± 5.3	5/3	2/6
Moderate stroke	56 ± 8.7	82.1 ± 60.0	36.1 ± 7.0	6/2	4/4
Severe stroke	61.8 ± 10.0	174.8 ± 94.7	17.5 ± 3.8	3/5	3/5
Healthy control	63.2 ± 7.6	-	-	4/2	-

M, male; F, female. R, right; L, left.

### 2.2. Experimental protocol

**Figure 1:**
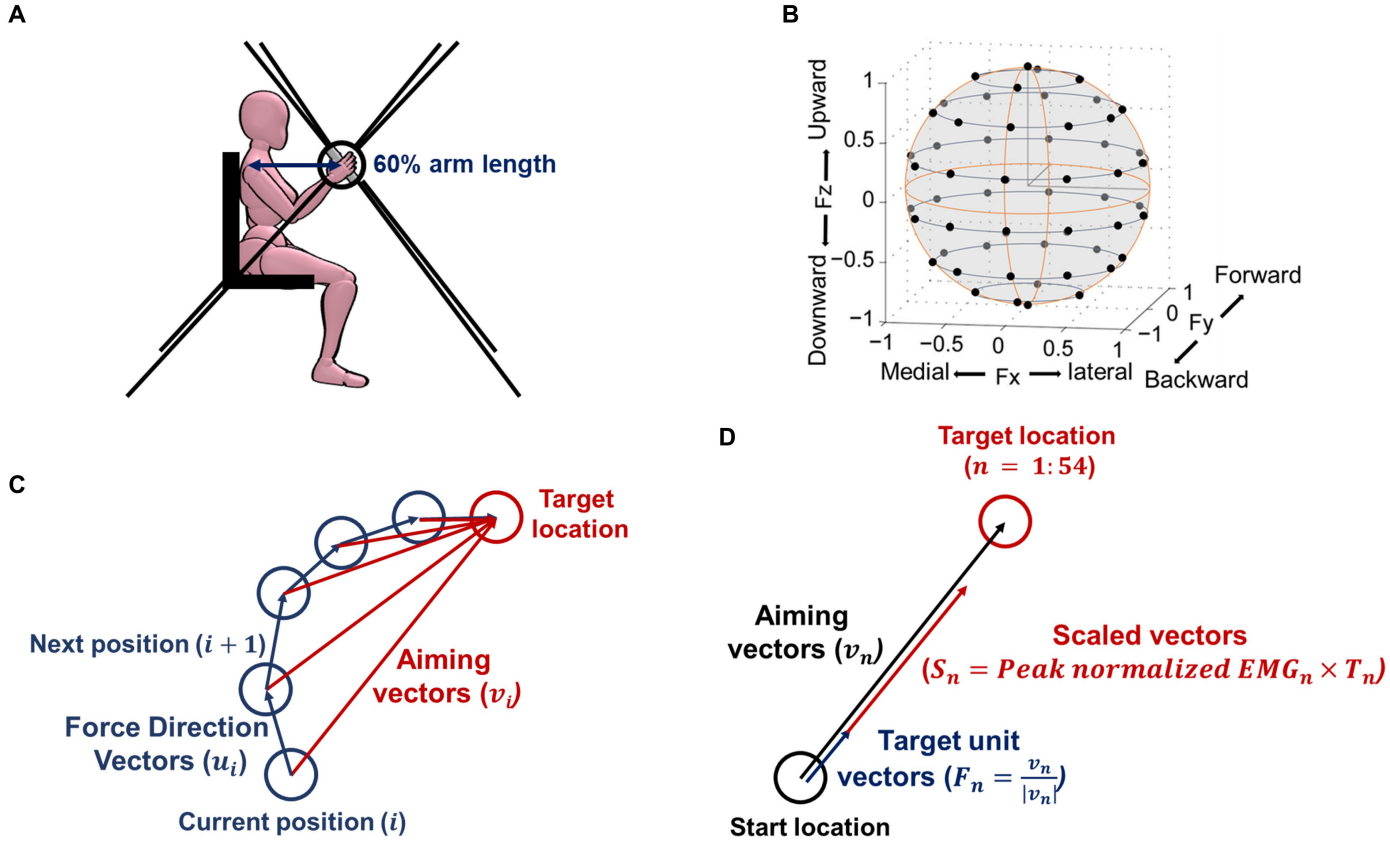
Isometric force measurement setup, target force location, and definition of vectors for calculating outcome measurements. **(A)** The side view of the experimental setup. Three-dimensional (3D) forces were recorded at the end-effector (represented as a bar in the picture) of the MACARM cable-robot. Participants’ seating position was adjusted to align the hand directly to the front of the ipsilateral shoulder at 60% of the arm length. The eight black lines represent the cables of the robot, attached to the eight motors (not shown), located at the corner of a cubic, which controls the location of the end-effector. **(B)** Black circles indicate 54 normalized target locations, equally distributed in the 3D force space. **(C)** An exemplary cursor movement to define the force direction and aiming vectors. Force direction vectors ( $u_{i}$ ) start at the current position towards the next position at the *i*^th^ moment, while aiming vectors ( $v_{i}$ ) start at the current position towards the target location at the *i*^th^ moment. **(D)** The definition of vectors for calculating a preferred direction. The 54 targets as unit vectors ( $F_{n}$ ), which represented the direction of the 54 force targets, were scaled by the peak normalized EMG magnitude of each muscle at each force target direction ( $S_{n}$ ). The linear summation of all these scaled vectors ( $S_{n}$ ) determined the preferred direction of each muscle activation.

Participants performed an isometric force target match task using the Multi-Axis Cartesian-based Arm Rehabilitation Machine [MACARM (5); Figure 1A]. Participants’ seating position was adjusted to align the hand directly to the front of the ipsilateral shoulder at a distance of 60% of arm length. Bracing and strapping were used to minimize the wrist and trunk movements while performing the task. After finalizing the participant’s position correctly, the participant had a short training session to understand how to perform the task. Participants controlled the location of the visual cursor on the screen using the endpoint force at the hand and matched the visual target that appeared on the screen. Each participant attempted to match 54 targets, in total, equally distributed in three-dimensional (3D) space (Figure 1B). For each trial, participants were instructed to relax in the baseline period (2 s), then voluntarily generate the force to match the target (isometric reaching period) once the target appeared. After the target match, they maintained the force constantly, confirmed by the visual cursor remaining within a target sphere (holding period; 0.8 s). A target force magnitude was 40% of the maximum lateral force (28). After the baseline period, age-matched control and stroke participants should complete each trial within 7 and 9 s, respectively. Three attempts per target were allowed if a target match failed within the time window. If successful, participants proceeded to the next target given in a random sequence. The age-matched control group performed the task with both arms to test any laterality of the composition of muscle synergies. The stroke group only performed the task with the contralesional arm.

### 2.3. Electromyographical and force data acquisition

Surface EMG signals were recorded at 1,920 Hz using a Bagnoli eight-channel surface EMG system (Delsys Incorporated, Natick, MA, United States) from eight muscles of the upper extremity: brachioradialis (BRD); biceps brachii (BIC); triceps brachii, long and lateral heads (TRIlong and TRIlat, respectively); deltoid, anterior, middle, and posterior fibers (AD, MD, and PD, respectively); and pectoralis major (clavicular fibers; PECTclav). Simultaneously, 3D endpoint force at the hand was collected at 64 Hz using MACARM.

### 2.4. Data analysis

Three-dimensional endpoint forces were filtered with a 4th-order Butterworth low-pass filter with a cut-off frequency of 6 Hz. A force onset was defined as the first point that exceeded 10% of the maximum first derivative of the force.

We computed five motor behavior parameters based on the processed endpoint force trajectories in the isometric reaching period: force tuning, number of peak speeds, trial completion duration, path length, and mean speed. The force tuning metric reflects the instantaneous force direction adjustment toward the target direction. The number of peak speeds reflects the smoothness of endpoint force generation. Trial completion duration, path length, and mean speed reflect temporal and spatial efficiency. The first parameter, the force tuning, was obtained as follows:


(1) $$\overset{k-1}{\underset{i=1}{\sum }}\frac{u_{i}.v_{i}}{\left|u_{i}\right|\left|v_{i}\right|}$$

where $v_{i}$ is the aiming vector (starting from the current position to the target location), $u_{i}$ is the force direction vector (starting from the current position to the next actual movement position) at each moment, and k is the number of data at each target (Figure 1C). The second parameter, the number of peak speeds, quantified the count of peaks observed in the endpoint force’s first derivative throughout the isometric reaching period. The third parameter, the trial completion duration, represented the total time to complete the trial. The fourth parameter, the path length, measured the accumulated distance covered by the endpoint force trajectories. The magnitude of the target force further normalized the accumulated distance. Lastly, the mean speed parameter was determined by finding the average value of the first derivative of the endpoint force.

All parameters were then transformed into a standard value to present relative to the age-matched control (29). The five metrics of the age-matched control group from all participants across 54 targets were transformed to a standard normal distribution using the Box-Cox equation. The coefficient of the Box-Cox equation, acquired from the data of the age-matched control group, was used to transform the five corresponding metrics of the stroke group. Ultimately, the five metrics were presented as Z-scores.

EMG data were preprocessed for further analysis. The electrocardiogram (ECG) noise was filtered from the PECTclav EMG signal using the wavelet transform decomposition (30). A DC component was removed by subtracting the mean of each muscle’s EMG signal from the ECG-filtered EMG. Then, a full wave rectification was applied. Baseline EMG values at a resting period were subtracted from the rectified EMG signals collected from each muscle to remove any muscle tone at rest. After the baseline subtraction, any negative values were replaced by zero to meet the non-negativity constraint of the following synergy identification. The EMG envelope was computed by 4th-order Butterworth low-pass filtering with a cut-off frequency of 10 Hz. Unit-variance normalization was applied to minimize intersubject variability for preferred direction calculation and prevent any bias towards high-variance muscle activation for synergy identification.

We calculated the preferred direction of individual muscle activation recorded during 54 force target matches under isometric conditions. The 54 targets’ unit vectors ( $F_{n}$ ), which represented the direction of the 54 force targets, were scaled by the peak normalized EMG magnitude ( $S_{n}$ ) of each muscle at each force target direction (Figure 1D). The linear summation of all these scaled vectors determined the preferred direction of each muscle per participant. The preferred direction vectors had components defined in the Cartesian coordinates. The X, Y, and Z components of the preferred direction were interpreted as the laterality of the lateral (+) - medial (−) axis, forward (+) - backward (−) axis, and upward (+) - downward (−) axis, respectively. In order to assess to what extent each muscle was activated in each of the six directions (lateral, medial, forward, backward, upward, and downward), we calculated the following:


(2) $$\frac{\sum S_{nx}}{\mathrm{Number}\;\mathrm{of}\;\mathrm{targets}}\;\left(\mathrm{Lateral}\;\mathrm{if}\;F_{nx}>0,\mathrm{Medial}\;\mathrm{if}\;F_{nx}<0\right)$$

(3) $$\frac{\sum S_{ny}}{\mathrm{Numb}er\;\mathrm{of}\;\mathrm{targets}}\;\left(\mathrm{Forward}\;\mathrm{if}\;F_{ny}>0,\mathrm{Backward}\;\mathrm{if}\;F_{ny}<0\right)$$

(4) $$\frac{\sum S_{nz}}{\mathrm{Number}\;\mathrm{of}\;\mathrm{targets}}\;\left(\mathrm{Upward}\;\mathrm{if}\;F_{nz}>0,\mathrm{Downward}\;\mathrm{if}\;F_{nz}<0\right)$$

where, $S_{nx},S_{ny}\text{,}$ and $S_{nz}$ are x, y, and z components of the n^th^ target scaled vector, respectively. $F_{nx},F_{ny}$ , and $F_{nz}$ are the n^th^ target unit vector’s x, y, and z components (Figure 1D).

To identify muscle synergies, we applied a non-negative matrix factorization (NMF) method to the EMG data (31). This technique can reduce the dimensionality of EMGs by reconstructing them as a linear combination of synergy vectors and their corresponding activation profiles. At each number of muscle synergies, ranging from one to the total number of muscles, muscle synergy extraction was repeated 100 times with random initial values to avoid a local minimum error. The set of muscle synergies that yielded the highest global Variance Account For (gVAF) was selected as a representative set for each number of muscle synergies. To determine the appropriate number of muscle synergies, we considered how the muscle synergies explained the total variation in all EMGs data (gVAF >90%) (32), in individual muscle EMG data (mVAF >60%), and the difference in gVAF when one additional number of muscle synergies was added (diffVAF <5%).

To compare the composition of each synergy vector between stroke and control, we quantified the similarity of muscle synergy vectors between stroke and control groups by calculating their scalar products. Regarding the activation profile of muscle synergy, we made the activation profile comparable between stroke and control groups with the same set of muscle synergy vectors. We fed the set of healthy synergy vectors, the synergy vectors averaged across control participants and represented as unit vectors, into an individual’s EMG data to calculate the corresponding activation profile. We then calculated the similarity of activation profiles using Pearson correlation between stroke and control groups.

### 2.5. Statistical analysis

We employed the Kruskal-Wallis test with a significance level of 5% to assess the statistical differences in median values among control, mild, moderate, and severe stroke groups within each muscle at each direction. This test is suitable when the assumptions for parametric tests are not met. Additionally, to determine which specific pairs of groups showed statistically significant differences, we applied a *post hoc* Tukey–Kramer multiple comparison test. To quantify the degree of the association among the laterality of the preferred direction of muscle activation, synergy similarity, FMA-UE score, and motor performance metrics, we utilized Spearman’s correlation coefficient. This non-parametric measure evaluates the strength and direction of monotonic relationships between variables. In our analysis, a significance level of 5% was used for all statistical tests, ensuring that results with *p*-values below this threshold were considered statistically significant.

## 3. Results

### 3.1. Alterations in the preferred direction of individual muscle activation and their association with the severity of motor impairment after stroke

**Figure 2:**
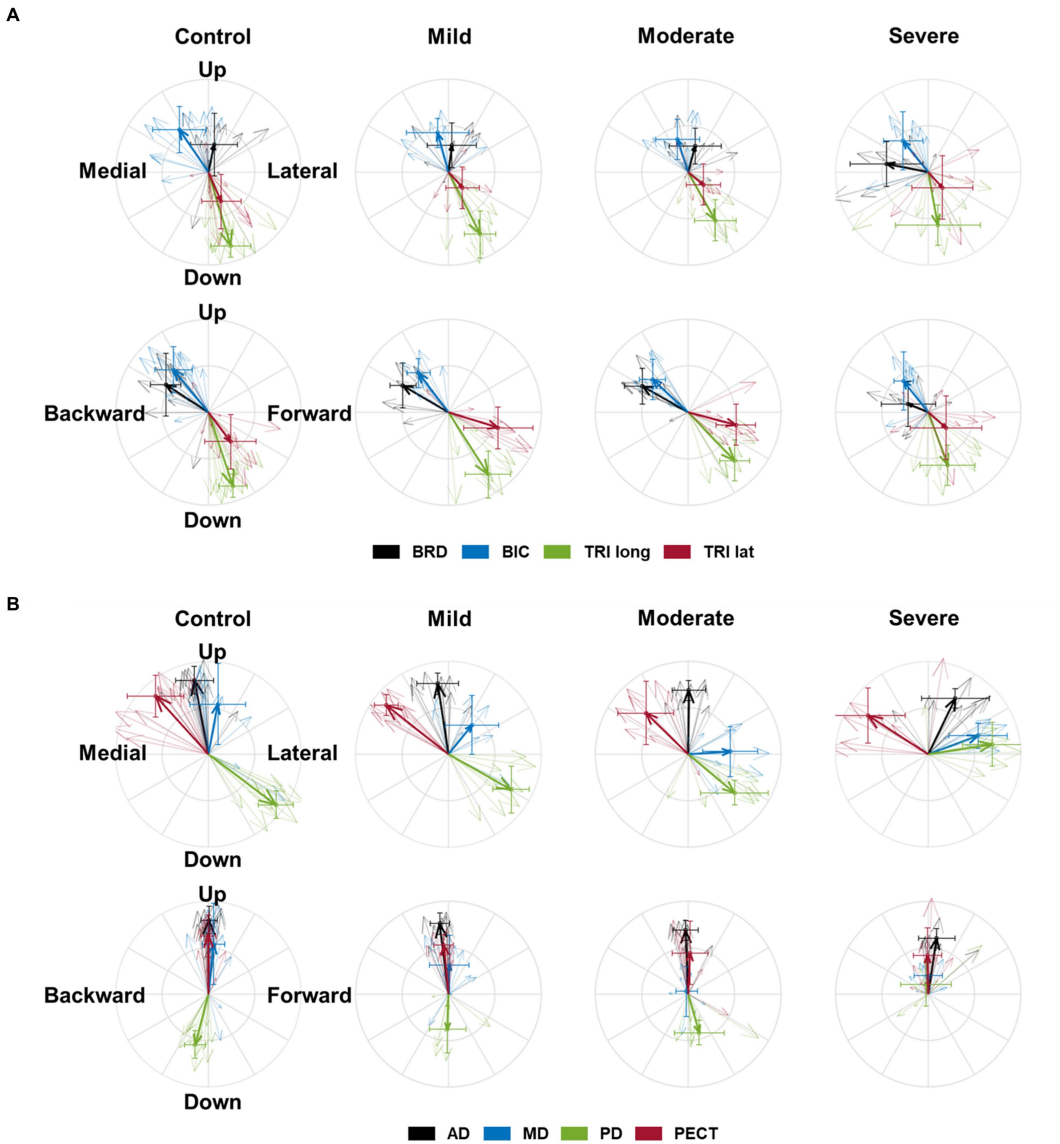
The preferred direction of individual muscles’ activation in health and stroke. **(A)** The preferred directions of four elbow muscles [brachioradialis (BRD); biceps brachii (BIC); triceps brachii, long and lateral heads (TRIlong and TRIlat, respectively)]. **(B)** The preferred directions of four shoulder muscles (the anterior (AD), middle (MD), and posterior (PD) deltoids; pectoralis clavicular fiber (PECT)). The direction of the vector indicates the preferred direction. The magnitude of each vector component is the laterality of the preferred direction on each axis. Thick arrows represent the mean (*n* = 8 in each sub-stroke group; *n* = 6 (participants) × 2 (bilateral arms) = 12 datasets in the healthy control) of preferred direction across individuals in each group, and thin arrows represent an individual’s preferred direction. The error bar indicates one standard deviation of each group on each axis.

The preferred direction of individual muscle activation was altered in isometric force generation after stroke, especially with severe impairment. Among the elbow muscles, the preferred direction of BRD was only altered in stroke with severe impairment, while the preferred directions of other elbow muscles were preserved post-stroke (Figure 2A). In age-matched control, the preferred direction of BRD was towards the upward and backward directions, while it changed towards the medial direction and the strength of the preference in the backward and upward directions decreased in severe stroke (Figure 2A). It was because the BRD in severely impaired stroke was significantly more activated in the medial direction compared to other groups, as shown in Table 2 ( $\chi^{2}=15.33,p<0.01$ ; as a *post hoc* analysis, allp<0.05 in severe vs. control, mild, or moderate). However, the BRD activation magnitude was not statistically different in the lateral direction compared to other groups ( $\chi^{2}=4.41,p=0.22$ ). Similarly, the BRD was significantly more involved in both forward ( $\chi^{2}=9.88,p<0.05$ ; severe vs. mild, p<0.05) and backward ( $\chi^{2}=10.54,p<0.05$ ; severe vs. mild, p<0.05 ) directional force generation in severe stroke compared to mild stroke (Table 2). However, the laterality of the preferred direction of BRD activation in the forward/backward axis decreased due to the more significant increments of BRD activation in the forward direction than the backward (Figure 2A). Moreover, significantly greater BRD activation was observed in both upward ( $\chi^{2}=12.53,p<0.01\text{;}$ severe vs. mild, p<0.05 ) and downward ( $\chi^{2}=7.91,p<0.05$ ; severe vs. mild, p<0.05) directional force generation in severe stroke compared to mild stroke (Table 2). The laterality of the preferred direction of BRD activation in the upward/downward axis decreased because of the more significant increments of BRD activation in the downward direction than the upward (Figure 2A).

**Table 2:** Group differences in muscle activation (mean ± std) to six orthogonal directions in 3D space.

	Control	Mild	Moderate	Severe	Control	Mild	Moderate	Severe	Control	Mild	Moderate	Severe
	Lateral				Forward				Upward			
BRD	2.10 ± 0.41	1.67 ± 0.39	2.12 ± 0.65	2.07 ± 0.65	1.40 ± 0.50	1.05 ± 0.28	1.37 ± 0.57	**2.44 ± 0.85** ^†^	2.67 ± 0.55	2.31 ± 0.37	2.71 ± 0.55	**3.27 ± 0.64** ^†^
BIC	1.06 ± 0.60	1.54 ± 0.42	1.86 ± 1.16	1.51 ± 0.52	0.84 ± 0.32	1.17 ± 0.38	1.39 ± 1.26	1.47 ± 0.57	2.11 ± 0.55	2.34 ± 0.35	2.65 ± 1.18	2.62 ± 0.63
TRIlong	1.74 ± 0.67	2.24 ± 0.80	2.56 ± 0.96	2.25 ± 0.41	1.65 ± 0.49	2.17 ± 0.57	2.55 ± 1.00	2.31 ± 0.87	0.82 ± 0.40	1.28 ± 0.84	1.84 ± 1.37	1.74 ± 0.87
**TRIlat**	2.06 ± 0.39	2.01 ± 0.41	2.58 ± 0.87	2.20 ± 0.43	2.18 ± 0.21	2.41 ± 0.47	2.87 ± 0.56	2.27 ± 0.47	1.95 ± 0.71	1.88 ± 0.64	2.61 ± 0.99	2.31 ± 0.71
AD	0.97 ± 0.77	1.15 ± 0.74	1.55 ± 0.64	**1.60 ± 0.42** ^*^	1.10 ± 0.44	1.07 ± 0.34	1.40 ± 0.68	1.50 ± 0.98	2.53 ± 0.49	2.55 ± 0.29	2.96 ± 0.62	2.60 ± 0.86
MD	1.83 ± 0.82	2.73 ± 0.64	**2.84 ± 0.46** ^*^	2.49 ± 0.26	1.64 ± 0.48	2.13 ± 0.77	1.96 ± 0.57	1.67 ± 0.72	2.84 ± 0.50	3.08 ± 0.93	2.60 ± 1.13	2.30 ± 0.77
PD	2.37 ± 0.36	2.82 ± 0.39	2.96 ± 0.86	2.19 ± 0.61	1.03 ± 0.23	**1.65 ± 0.68** ^*^	2.15 ± 1.03	1.25 ± 0.79	0.82 ± 0.39	1.52 ± 0.63	2.04 ± 1.31	1.63 ± 0.93
PECT	0.63 ± 0.28	1.37 ± 0.67	1.61 ± 1.34	1.08 ± 0.65	1.20 ± 0.30	1.87 ± 0.61	1.92 ± 1.15	1.82 ± 0.76	2.33 ± 0.33	2.95 ± 0.71	3.03 ± 1.00	2.69 ± 0.90
	Medial				Backward				Downward			
BRD	−1.95 ± 0.62	−1.57 ± 0.41	−1.90 ± 0.64	**−2.94 ± 054** ^*,†,‡^	−2.82 ± 0.46	−2.51 ± 0.32	−2.99 ± 0.54	**−3.19 ± 0.52** ^†^	−1.96 ± 0.73	−1.60 ± 0.40	−1.90 ± 0.63	**−2.77 ± 0.73** ^†^
BIC	−1.82 ± 0.44	−1.81 ± 0.57	−2.11 ± 1.16	−2.01 ± 0.59	−1.96 ± 0.58	−2.19 ± 0.47	−2.71 ± 1.19	−2.30 ± 0.52	−1.01 ± 0.41	−1.30 ± 0.44	−1.69 ± 1.54	−1.62 ± 0.48
TRIlong	−1.17 ± 0.38	−1.39 ± 0.61	−1.78 ± 1.09	−1.73 ± 0.76	−1.23 ± 0.33	−1.41 ± 0.82	−1.73 ± 1.06	−1.89 ± 0.75	−2.73 ± 0.34	−2.87 ± 0.48	−2.93 ± 0.80	−2.94 ± 0.66
TRIlat	−1.73 ± 0.48	−1.63 ± 0.55	−2.11 ± 0.69	−1.62 ± 0.70	−1.91 ± 0.76	−1.41 ± 0.75	−2.07 ± 1.00	−1.96 ± 0.93	−2.72 ± 0.40	−2.26 ± 0.40	−2.77 ± 0.65	−2.54 ± 0.73
AD	−1.33 ± 0.84	−1.41 ± 0.33	−1.51 ± 0.74	**−0.78 ± 0.36** ^†^	−1.26 ± 0.54	−1.50 ± 0.49	−1.77 ± 0.67	−1.27 ± 0.24	−0.61 ± 0.71	−0.69 ± 0.47	−1.16 ± 0.79	−0.81 ± 0.35
MD	−1.59 ± 0.45	−2.09 ± 0.82	−1.69 ± 0.85	**−0.91 ± 0.41** ^†^	−1.75 ± 0.55	−2.45 ± 0.75	−2.40 ± 0.85	−1.75 ± 0.33	−1.54 ± 0.89	−2.31 ± 0.88	−2.38 ± 0.87	−1.54 ± 0.48
PD	−0.62 ± 0.40	−1.17 ± 0.60	−1.71 ± 1.15	**−0.38 ± 0.18**^†^,^‡^	−1.56 ± 0.40	−1.94 ± 0.35	−2.26 ± 0.94	−1.30 ± 0.44	−2.14 ± 0.40	−2.42 ± 0.75	−2.90 ± 1.18	**−1.10 ± 0.42** ^*,†,‡^
PECT	−1.99 ± 0.60	−2.97 ± 0.69	−2.69 ± 1.06	−2.55 ± 0.86	−1.39 ± 0.24	−2.30 ± 0.70	−2.31 ± 1.16	−1.94 ± 0.68	−0.82 ± 0.37	−1.65 ± 0.77	−1.78 ± 1.52	−1.36 ± 0.77

BRD, brachioradialis; BIC, biceps brachii; TRIlong, triceps brachii, long; TRIlat, triceps lateral heads; AD, deltoid anterior fibers; MD, deltoid middle fibers; PD, deltoid posterior fibers; PECTclav, pectoralis major clavicular fibers. ^*^, ^†^, and ^‡^, any comparison with age-matched, mild, and moderate groups, respectively (all *p* < 0.05).

Regarding shoulder muscles, we found alterations in the preferred directions of AD, MD, and PD activations during isomeric force generation after stroke. AD was significantly activated more in the lateral direction ( $\chi^{2}=9.62,p<0.05\text{;}$ severe vs. mild, p<0.05) and less in the medial direction ( $\chi^{2}=9.86,p<0.05$ ; severe vs. mild, p<0.05) in severe stroke compared to mild stroke (Table 2). The alterations resulted in a change in the preferred direction of AD, which was towards the medial direction in age-matched control, towards the lateral direction in severe stroke (Figure 2B). Meanwhile, MD did not prefer medial-lateral directional force generation in the age-matched control. However, the preferred direction of MD activation in severe stroke was altered towards the lateral direction (Figure 2B). Table 2 shows this alteration in severe stroke due to the significantly less involvement in the medial directional force generation compared to mild stroke ( $\chi^{2}=11.64,p<0.01$ ; severe vs. mild, p<0.05 ). Lastly, the preferred direction of PD activation in the severe stroke was changed from downward to upward (Figure 2B). This alteration was related to significantly less activation of PD in the downward directional force generation ( $\chi^{2}=9.10,p<0.05$ ) compared to other groups, as shown in Table 2 (severe vs. control, mild, or moderate, all p<0.05 ). Overall, these alterations resulted in the preferred directions of AD, MD, and PD activation to get closer to each other in severe stroke (Figure 2B).

These alterations in the preferred direction were associated with the severity of motor impairment after stroke. Among the elbow muscles, the laterality of the preferred directions of BRD in all three axes was significantly correlated with the FMA-UE score (L-M, r=0.49,p<0.05 ; F-B, r=−0.64,p<0.05 ; U-D, r=0.44,p<0.05 , Table 3). As the FMA-UE score decreased, the preferred direction of BRD was towards the medial, forward, and downward directions (Table 3). Also, alterations of the preferred directions in all shoulder muscles were significantly associated with motor impairments ( p<0.05 , Table 3). As the FMA-UE score decreased, the preferred direction of AD was more towards the lateral ( r=−0.55,p<0.05 ) and forward directions ( r=−0.50,p<0.05 ), and the preferred directions of MD and PD were more towards the lateral ( r=−0.55,p<0.05) and upward directions ( r=−0.60,p<0.05) , respectively (Table 3).

**Table 3:** The Spearman’s rank correlation coefficient between motor impairment and laterality of the preferred direction of muscle activation.

	L-M	F-B	U-D
BRD	**0.49** ^*^	**−0.64** ^**^	**0.44** ^*^
BIC	−0.05	−0.29	0.11
TRIlong	0.34	0.25	−0.30
TRIlat	0.02	0.25	−0.28
AD	**−0.55** ^**^	**−0.50** ^*^	0.19
MD	**−0.55** ^**^	−0.11	−0.21
PD	−0.35	−0.07	**−0.60** ^**^
PECT	0.10	−0.24	−0.01

L-M, Lateral-Medial axis; F-B, Forward-Backward axis; U-D, Upward-Downward axis; BRD, brachioradialis; BIC, biceps brachii; TRIlong, triceps brachii, long; TRIlat, triceps lateral heads; AD, deltoid anterior fibers; MD, deltoid middle fibers; PD, deltoid posterior fibers; PECTclav, pectoralis major clavicular fibers.

* *p* < 0.05.

** *p* < 0.01.

### 3.2. Alterations in the attributes of muscle synergy and their association with the severity of motor impairment after stroke

**Figure 3:**
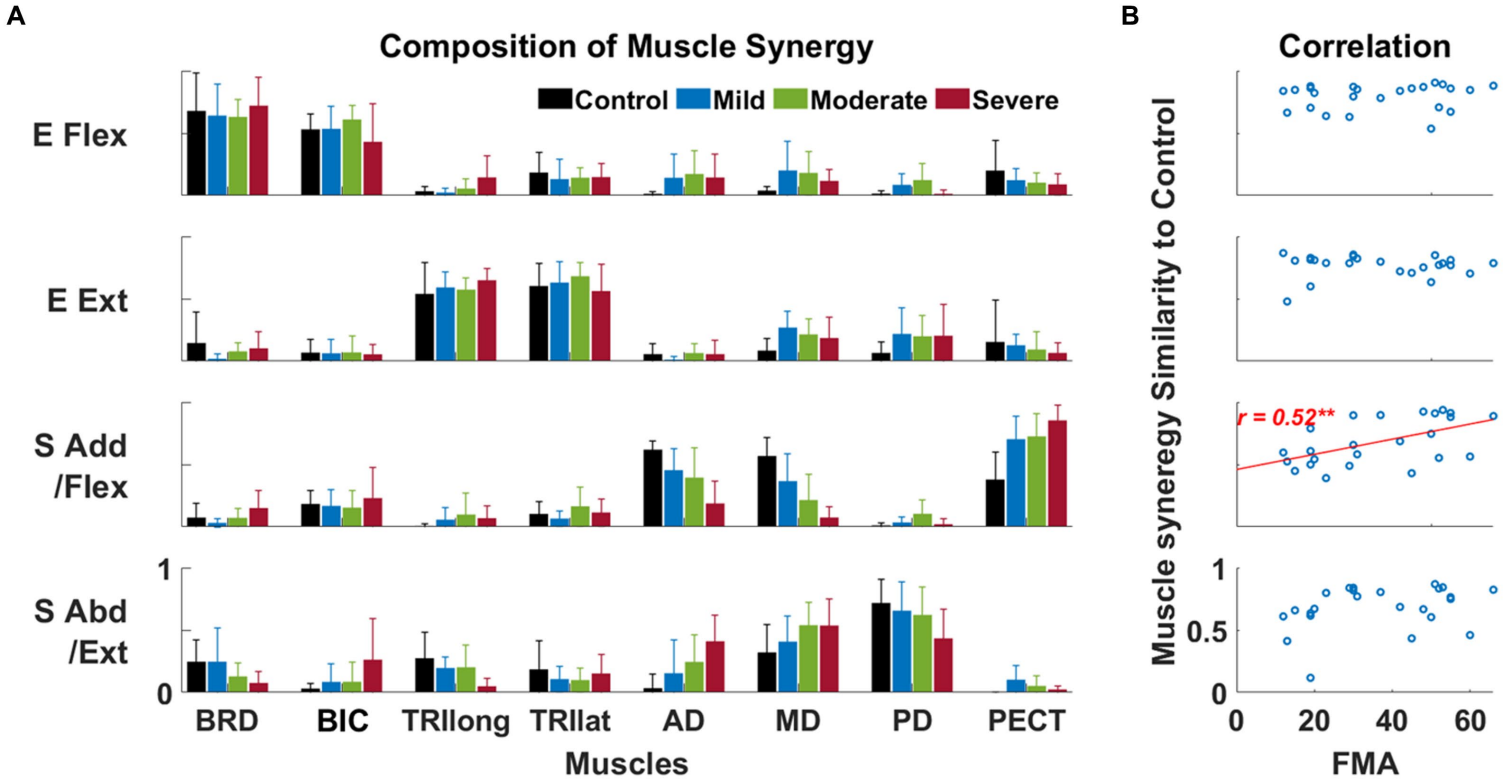
The composition of muscle synergy across groups and its relationship to the Fugl-Meyer assessment score. **(A)** Four identified muscle synergies in control and each of three stroke sub-groups. Each bar represents each muscle’s mean weight (*n* = 8) within a synergy for each group; one standard deviation of muscle weights is indicated as a bar on the mean value. Based on the composition of muscle synergy, the function of each synergy was defined, such as the elbow flexor (E Flex), the elbow extensor (E Ext), the shoulder adductor/flexor (S Add/Flex), and the shoulder abductor/extensor (S Abd/Ext). **(B)** The correlation between the similarity score of synergy composition between the control and stroke groups and the FMA-UE score. Each circle represents the data collected from a post-stroke individual. Only statistically significant correlation is presented with a correlation coefficient (Spearman’s rank correlation coefficient) and linear regression line (**, *p* < 0.01).

The alteration in the composition of the shoulder muscle synergy was associated with the severity of post-stroke motor impairment. Four synergies were identified in both healthy and stroke groups. Based on the major mechanical actions of significant muscle weights in each synergy, the function of each synergy was defined as an elbow flexor (E Flex), elbow extensor (E Ext), shoulder adductor/flexor (S Add/Flex), and shoulder abductor/extensor (S Abd/Ext), respectively. Among those synergies, we observed that S Add/Flex and S Abd/Ext synergies were altered across the level of motor impairment groups, whether the E Flex and E Ext synergies were preserved after stroke in all groups (Figure 3A). Though both shoulder synergies were altered after the stroke, we found that only the similarity score in S Add/Flex synergy was significantly correlated with the FMA-UE score ( r=0.52,p<0.01 , Figure 3B).

**Figure 4:**
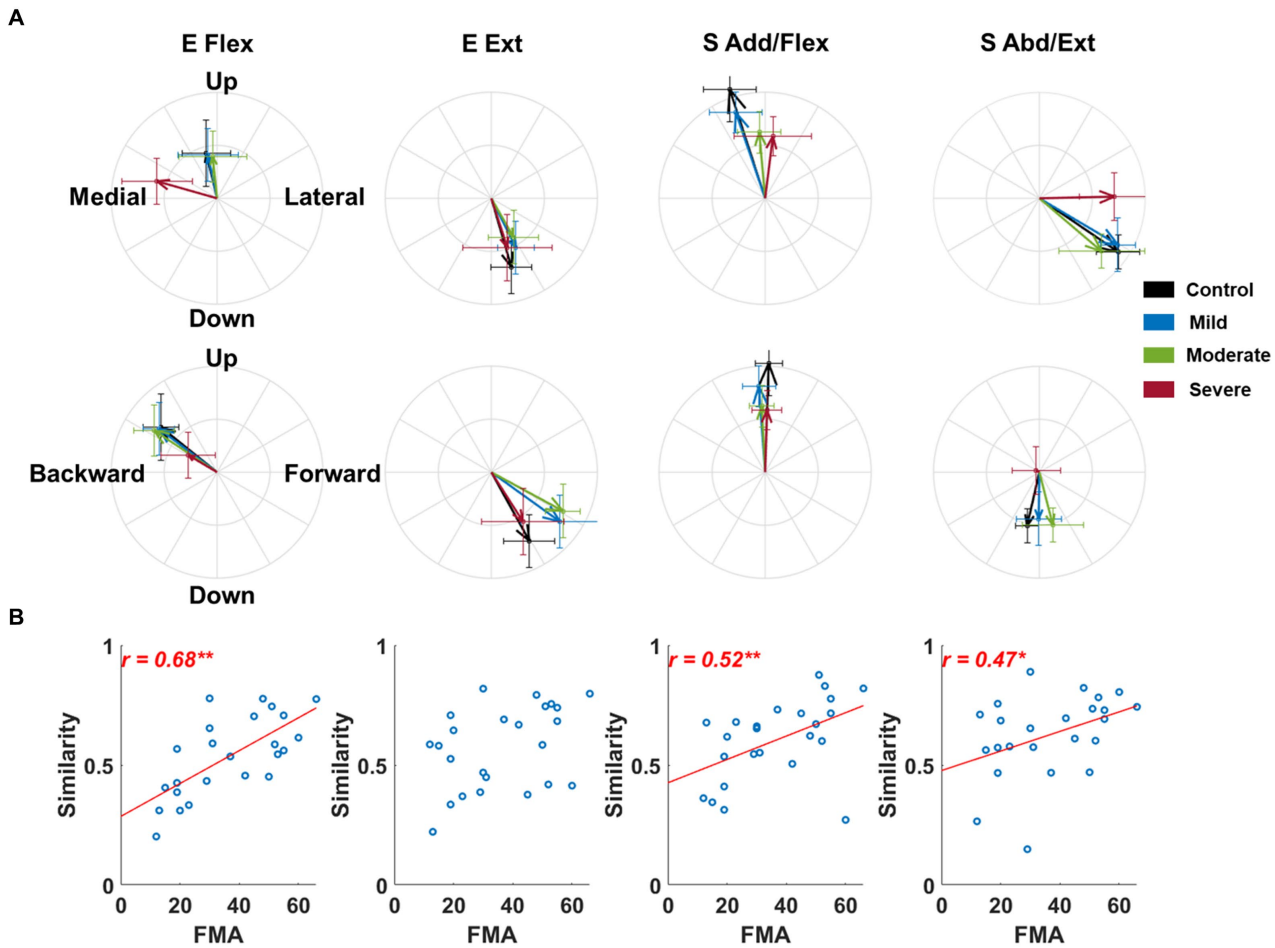
The preferred direction of synergy activation profiles across groups and its relationship to the Fugl-Meyer assessment score. **(A)** The preferred direction of synergy activation profiles. The direction of the vector indicates the preferred direction. The magnitude of each vector component is the laterality of the preferred direction on each axis. Thick arrows represent the mean (n = 8) of the preferred direction across individuals in each group. The error bar indicates one standard deviation of each group on each axis. **(B)** The correlation between the similarity of the activation profile to the healthy control and motor impairment post-stroke. Each circle represents the data of an individual post-stroke. Only statistically significant correlation is presented with a correlation coefficient and linear regression line. The *r*-value indicates Spearman’s rank correlation coefficient. The asterisk indicates a significant correlation (*, *p* < 0.05; **, *p* < 0.01).

The alteration in the activation profile of muscle synergy was associated with motor impairments except for elbow extensor synergy after stroke. In these results, we assumed that all post-stroke participants had the same synergy composition as the control to make a fair comparison of synergy activation profiles with the control group. Generally, the preference for the forward and backward direction of E Ext and E Flex synergy activation decreased in the stroke group of severe impairment, respectively (Figure 4A). In addition, the preferred direction of each synergy activation was not overlapped with each other in the frontal plane (Figure 4A). More specifically, though the composition of both E Flex and E Ext synergies was preserved in stroke, as we observed in the results of alterations in synergy composition, the preferred direction of E Flex synergy activation was shifted from a backward-upward direction to a medial direction (Figure 4A). Since the compositions of shoulder synergies were altered in stroke, thus if we assumed that stroke had the same synergy composition as the control group, we expected that stroke-induced alterations would be embedded within the activation profile. As we expected, the preferred direction of S Add/Flex was altered from medial-upward to lateral-upward direction, and the preferred direction of S Abd/Ext was also altered from lateral-downward to pure lateral direction. These alterations were associated with the FMA-UE score (E Flex, r=0.68,p<0.01 ; S Add/Flex, r=0.52,p<0.01 ; S Abd/Ext, r=0.47,p<0.05 , Figure 4B).

### 3.3. Association between alterations in the preferred direction of individual muscle and alterations in muscle synergy attributes after stroke

We found that alterations in synergy attributes associated with the severity of impairment correlated with changes in the preferred direction of individual muscles, which were also related to the severity of impairment. In the composition of the synergy vector, the similarity of S Add/Flex decreased as the preferred directions of AD and MD were redirected in a more lateral direction (AD, r=−0.44,p<0.05 ; MD, r=−0.41,p<0.05 , Table 4), and that of PD was redirected in a more upward direction ( r=−0.49,p<0.05 , Table 4). Regarding the activation profile, Table 4 shows that the similarity of E Flex decreased when the preferred direction of BRD was redirected in a more medial ( r=0.44,p<0.05 ), forward ( r=−0.72,p<0.05 ), and downward direction ( r=0.58,p<0.05 ). The similarity of S Add/Flex decreased as the preferred direction of AD and MD were redirected in a more lateral direction (AD, r=−0.58,p<0.05 ; MD, r=−0.58,p<0.05 , Table 4). The similarity of S Abd/Ext decreased as the preferred direction of PD was redirected in a more upward direction ( r=−0.71,p<0.05 , Table 4).

**Table 4:** The Spearman’s rank correlation coefficient between the laterality of preferred direction of individual muscle activation and the synergy similarity.

	Synergy composition similarity														
		E Flex	E Ext	S Add/Flex	S Abd/Ext		E Flex	E Ext	S Add/Flex	S Abd/Ext		E Flex	E Ext	S Add/Flex	S Abd/Ext
BRD	Lateral-Medial	−0.10	0.049	0.36	**0.60** ^**^	Forward-Backward	−0.32	0.09	**−0.45** ^*^	−0.23	Upward-Downward	0.34	0.15	0.05	0.06
BIC		−0.30	0.10	−0.03	0.25		−0.31	−0.13	−0.20	−0.10		0.08	0.03	−0.02	0.07
TRIlong		0.23	−0.22	**0.43** ^*^	0.10		**0.61** ^**^	0.40	**0.50** ^*^	**0.45** ^*^		−0.25	−0.16	**−0.54** ^**^	−0.29
TRIlat		0.23	−0.12	0.02	−0.29		0.40	**0.48** ^*^	**0.60** ^**^	**0.52** ^*^		−0.40	−0.38	**−0.54** ^**^	**−0.41** ^*^
AD		−0.06	−0.09	−0.44^*^	−0.45^*^		0.04	0.25	−0.16	−0.05		0.09	0.16	0.15	0.24
MD		0.004	0.08	−0.41^*^	−0.51^*^		0.22	**0.47** ^*^	0.05	0.18		0.04	0.03	−0.06	−0.06
PD		0.27	0.15	−0.13	−0.32		−0.11	0.37	−0.13	0.25		−0.19	0.12	**−0.49** ^*^	−0.32
PECT		−**0.47**	−0.36	−0.04	−0.01		−0.02	−0.17	−0.12	−0.30		0.27	0.24	**0.51** ^*^	0.02
	Synergy Activation Similarity														
		E Flex	E Ext	S Add/Flex	S Abd/Ext		E Flex	E Ext	S Add/Flex	S Abd/Ext		E Flex	E Ext	S Add/Flex	S Abd/Ext
BRD	Lateral-Medial	**0.44** *	0.25	0.51	−0.01	Forward-Backward	**−0.72** ^**^	**−0.52** ^*^	**−0.47** ^*^	**−0.51** ^*^	Upward-Downward	**0.58** ^**^	0.16	−0.05	0.23
BIC		0.01	−0.07	0.18	−0.27		**−0.53** ^**^	−0.33	−0.27	**−0.41** ^*^		**0.41** ^*^	−0.04	0.22	0.22
TRIlong		**0.41** ^*^	**0.41** ^*^	0.22	**0.42** ^*^		0.36	**0.75** ^**^	0.27	0.33		−0.26	**−0.77** ^**^	**−0.49** ^*^	**−0.65** ^**^
TRIlat		0.13	0.21	−0.22	0.20		0.24	**0.74** ^**^	**0.48** ^*^	0.13		−0.16	**−0.87** ^**^	**−0.43** ^*^	−0.38
AD		−0.33	−0.36	−**0.58+**	0.00		**−0.47** ^*^	0.06	−0.26	0.09		0.15	0.28	**0.64** ^**^	0.14
MD		−0.27	−0.20	−0.58^**^	0.03		−0.03	0.26	0.22	−0.16		−0.23	−0.27	0.06	**−0.48** ^*^
PD		−0.06	0.13	−0.22	0.32		0.00	0.00	−0.10	**−0.56** ^**^		**−0.54** ^**^	**−0.53** ^**^	−0.37	**−0.71** ^**^
PECT		−0.09	−0.51	−0.12	−0.45^*^		−0.37	−0.08	−0.31	−0.17		0.35	0.39	0.21	0.22

BRD, brachioradialis; BIC, biceps brachii; TRIlong, triceps brachii, long; TRIlat, triceps lateral heads; AD, deltoid anterior fibers; MD, deltoid middle fibers; PD, deltoid posterior fibers; PECTclav, pectoralis major clavicular fibers.; E Flex, elbow flexor; E Ext, elbow extensor; S Add/Flex, shoulder adductor/flexor; S Abd/Ext, shoulder abductor/extensor.

* *p* < 0.05.

** *p* < 0.01.

### 3.4. Association between qualities of isometric motor performance and either alteration in the preferred direction of individual muscle activation or muscle synergy attributes after stroke

**Figure 5:**
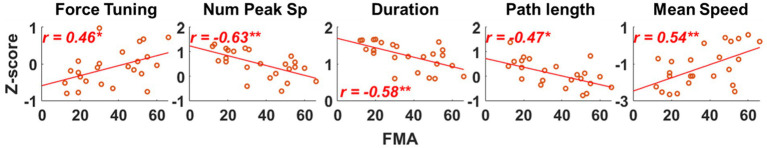
Evaluating the association between qualities of isometric motor performance and motor impairment after stroke. Five qualities of motor performance measures were evaluated: Force tuning, Number of peak speeds (Num Peak Sp), Duration, Path length, and Mean Speed. Each red circle represents a post-stroke individual. Only statistically significant correlation is presented with a correlation coefficient and linear regression line. The r-value indicates Spearman’s rank correlation coefficient. The asterisk indicates a significant correlation (*, *p* < 0.05; **, *p* < 0.01).

We found that as the severity of motor impairment increased, the qualities of isometric motor performance decreased, and these decreased qualities of motor performance post-stroke were associated with alterations in the preferred direction of individual muscle activation. Figure 5 shows that as the motor impairment increased, the stroke participants tended to have more difficulty controlling the force toward the target force direction (Force tuning, r=0.46,p<0.05 ). They also performed the isometric force target matches with a less smooth trajectory and slower force generation (Num Peaks Sp, r=−0.63,p<0.01 ; Mean Speed, r=0.54,p<0.01 ). Consequently, more time and unnecessary movements were observed post-stroke during isometric force generation (Duration, r=−0.58,p<0.01 ; Path Length, r=0.47,p<0.05 ). The decreased quality of the isometric motor performance was associated with alterations in the preferred direction of BRD and PD activation (Table 5). In particular, if the BRD activation was relatively more involved in the forward directional force generation than backward, all the qualities of motor performance decreased ( p<0.05 , Table 5). In addition, as the PD activation was relatively more involved in the upward directional force generation than downward, all the qualities of motor performance except the force tuning component decreased ( p<0.05 , Table 5). The examples of other alterations in the preferred direction of muscle activation related to motor impairment were BRD in the lateral/medial and upward/downward axes, AD in the lateral/medial and forward/backward axis, and MD in the lateral/medial axis (Table 2). However, these examples were not associated with the qualities of motor performance (Table 5).

**Table 5:** The Spearman’s rank correlation coefficient between qualities of motor behaviors and either the laterality of preferred direction or synergy similarity.

Laterality of preferred direction		Force tuning	Num peak speeds	Duration	Path length	Mean speed
BRD	Lateral-Medial	−0.05	−0.35	−0.36	−0.08	0.34
AD		−0.07	0.23	0.30	0.20	−0.38
MD		0.05	0.21	0.22	0.08	−0.40
PD		0.07	0.05	0.13	0.16	−0.03
BRD	Froward-Backward	−0.68^**^	0.61^**^	0.61^**^	0.72^**^	−0.43^*^
AD		−0.16	0.21	0.25	0.23	−0.35
MD		−0.09	0.15	0.23	0.08	−0.13
PD		−0.04	00.08	0.03	−0.18	−0.30
BRD	Upward-Downward	0.07	−0.38	−0.38	−0.14	0.26
AD		0.06	0.07	0.15	−0.03	0.26
MD		−0.15	0.35	0.38	0.11	−0.47^*^
PD		−0.37	0.52^*^	0.53^**^	0.43^*^	−0.54^**^
Synergy composition similarity						
S Add/Flex		0.43^*^	−0.46^*^	−0.46^*^	−0.45^*^	0.41^*^
Synergy activation similarity						
E Flex		0.54^**^	−0.82^**^	−0.82^**^	−0.62^**^	0.56^**^
E Ext		0.55^**^	−0.48^*^	−0.41^*^	−0.49^*^	0.29
S Abd/Ext		0.53^**^	−0.56^**^	−0.50^*^	−0.40	0.53^**^

BRD, brachioradialis; BIC, biceps brachii; TRIlong, triceps brachii, long; TRIlat, triceps lateral heads; AD, deltoid anterior fibers; MD, deltoid middle fibers; PD, deltoid posterior fibers; PECTclav, pectoralis major clavicular fibers.; E Flex, elbow flexor; E Ext, elbow extensor; S Add/Flex, shoulder adductor/flexor; S Abd/Ext, shoulder abductor/extensor.

* *p* < 0.05.

** *p* < 0.01.

More interestingly, among the alterations in synergy composition and synergy activation profile in stroke, only the alterations associated with both the alteration of BRD activation in the forward/backward axis and PD in the upward/downward axis (Table 4) showed a significant relationship with the qualities of isometric motor performance ( p<0.05 ). Table 5 shows that these qualities of motor performance included force tuning, number of peaks speed, duration, path length, and mean speed, except in the cases of the associations between the activation profile of E Ext and mean speed as well as the activation profile of S Abd/Ext and path length.

## 4. Discussion

The current study aimed to understand how alterations in the preferred direction of individual muscles contribute to the overall motor deficits observed following stroke. Specifically, we examined the alterations in the preferred direction of individual muscle activation during isometric force generation after stroke and their relationship with intermuscular coordination, motor impairment, and motor performance using the three following factors: alterations in muscle synergy attributes, the FMA-UE scores, and force trajectory features. The post-stroke alterations in the preferred direction of BRD, AD, MD, and PD were correlated with the motor impairment level and attributed to the changes in muscle synergy characteristics. Only alterations in the preferred direction of BRD and PD activation in forward-backward and upward-downward axes were associated with the qualities of isometric force generation, respectively. These findings suggest that alterations in the preferred direction of individual muscle activation contribute to various aspects of motor deficit following stroke.

### 4.1. Co-contraction of antagonistic muscles at the elbow joint post-stroke

Previous studies have examined the co-contraction of antagonistic muscle pairs and its effects on voluntary movement after stroke. These studies have demonstrated that co-contraction is associated with clinical assessment scores and may contribute to restricting motor performance following stroke (13, 14, 16). For example, Chalard et al. investigated the co-contraction of muscles at the elbow joint during isometric elbow extension after stroke, finding an association between co-contraction and clinical assessment scores, such as those of FMA-UE and the Action Research Arm Test. Similarly, Chae et al. examined the co-contraction of the wrist joint during isometric wrist flexion and extension. They also found that co-contraction was correlated with the FMA-UE and the Arm Motor Ability Test scores, respectively. Consistent with these previous observations, our current findings indicated that the increased involvement of the brachioradialis (BRD), an elbow flexor, as an antagonistic muscle during forward directional force generation was negatively associated with the FMA-UE score (Table 3). In addition, our results showed that this alteration negatively affected isometric force control; we observed the negative relationship between the greater BRD activation magnitude in the forward directional force generation and the quality of isometric force control in terms of smoothness, spatial and temporal efficiency, and the ability to adjust the force toward the target (Table 5). While previous studies solely explored the relationship between co-contraction and clinical assessment scores (13, 14, 16), our study extends this understanding by establishing an association between muscle co-contraction and not only the clinical assessment scores but also the qualities of motor performance. Consequently, our results provide congruent evidence supporting the contribution of muscle co-contraction to motor performance limitations after stroke.

### 4.2. The potential relationship between alterations in the preferred direction of shoulder muscle activation and anti-gravitational support following stroke

Gravitational loading affects the reaching movement after a stroke. Beer et al. found that when stroke participants generated abduction and external torque at the shoulder to support against gravity actively, the speed and range of elbow extension movement were reduced (33). Another study showed that the preferred directions of shoulder muscles were shifted, favoring the shoulder abduction/adduction direction after stroke (3). In addition, the range of active reaching increased when stroke participants utilized additional external gravitational compensation, such as arm support or robot assistance (34, 35). Consistent with these previous findings, alterations in the preferred direction of PD towards the upward direction and those of AD, MD, and PD towards the lateral direction (Figure 2; Table 2) indicate that shoulder muscles are preferred to generate abduction torque, which may contribute to active anti-gravitational support in the arm following a stroke.

Prange et al. also showed that gravity compensation influences not only the level of activity on antigravity muscles directly, such as AD, but also other MD and PD, suggesting an indirect effect on the inappropriately coupled muscles after stroke (36). Our results support this idea that alterations in the preferred direction of shoulder muscles were correlated with changes in synergistic muscle groups in the shoulder (Table 4), inducing abnormal AD, MD, and PD co-activation. Moreover, these alterations in the preferred direction of shoulder muscles were associated with motor impairment assessed by FMA-UE (Table 3). In particular, the less involvement of PD in the downward directional force generation, which was the primary function of PD in age-matched controls, negatively impacted the qualities of isometric motor performance (Table 5). However, previous studies suggested that coupling shoulder abduction torque with simultaneous elbow flexion torque also played an important role in inducing deteriorated movement following a stroke (3, 37, 38). Our study indirectly supports this idea, showing that only the alterations in muscle synergy, correlated with both forward-backward and upward-downward preferred directional changes in BRD and PD, were associated with qualities of isometric motor performance (Tables 4, 5). These results indicate that stroke-induced alterations in the preferred direction of elbow flexor and shoulder abductor muscles likely have an essential role in motor performance.

### 4.3. Potential underlying mechanisms of alterations in the preferred direction of individual muscle activation after stroke

Our finding suggests that the alterations in the preferred direction of individual muscle activation might be associated with the underlying mechanism of intermuscular coordination impairment after stroke. A previous computational modeling study showed that, after removing the neurons with a specific preferred direction around the motor cortex area, the affected hemisphere would be reorganized and restore the directional motor command with supervised learning (39). However, with unsupervised learning, the reorganization would occur in a certain attempted way, which becomes maladaptive, increasing the representation of compensatory movements in neighboring neurons from damaged ones in the motor cortex area (39). Previous research revealed that the severe paresis group after stroke barely used the affected arm because the affected arm could not move against gravity (40). The desire to utilize the affected arm against gravity might induce maladaptive compensatory movements. From a biomechanical perspective, keeping the forearm posture close to the shoulder joint (e.g., wrist flexion and elbow flexion) may induce a more accessible arm lift using shoulder muscles because the center of mass of the arm can be closer to the shoulder joint. Furthermore, the brainstem pathway has been considered a possible residual descending motor pathway after stroke (41–43), known for controlling proximal muscles rather than distal muscles (44–46). Thus, we speculate that a compensatory movement to lift the arm against gravity would be related to alterations in the preferred direction of elbow and shoulder muscle activation after stroke considering the previous findings (1): a chance for maladaptive reorganization in the motor cortex area following compensatory motor attempts (2), using a biomechanically easier way to lift the affected arm against gravity resulting in abnormal, and (3) the residual descending motor pathway of proximal muscles. Further study will be needed to determine whether these alterations in the preferred direction occurred with external gravity compensation and how these alterations would be developed from an acute to the chronic stage after stroke onset.

### 4.4. Limitations

One of the limitations of this study is that we could not investigate the temporal features of muscle activation in intermuscular coordination. Temporal features like multiple muscles’ onset and offset timing would affect movement coordination. However, these temporal features would play a more important role in dynamic movement tasks and were outside the scope of the present study, which focused on spatial features of muscle activation under isometric conditions. Along with this limitation, the generalizability of results in this study to dynamic motor tasks would be questionable. In dynamic tasks, the biomechanical properties of muscle should change through movement, and the timing of muscle activation would be critical to coordinate the movement. Since the present study focuses on the effect of spatial features of muscle activation on intermuscular coordination, an isometric task would be more suitable to exclude these additional effects on intermuscular coordination.

### 4.5. Clinical implications

Some previous intervention studies have utilized surface EMG mapping to the visualized cursor movement to improve abnormal co-contraction or/and co-activation of upper limb muscles (25–27). Even though these studies showed their training effectiveness primarily based on metrics of muscle co-activation and/or clinical assessment scores, whether the intervention-targeted muscle activation improved from the perspective of true motor recovery (47, 48) was not clearly revealed. In addition, these studies did not specify the appropriate direction of movement to train the target muscle activation pattern. It might cause alternative ways to accomplish the training goal.

Our study provided the characteristics of the alteration in individual muscle activation directionality post-stroke in the upper extremity and how the changes were associated with alterations in intermuscular coordination, clinical motor impairment, and qualities of isometric motor performance post-stroke. We found that alterations in the preferred direction of individual muscles were related to the alterations in the activation profile of synergistic muscle groups after stroke; the changes in the preferred direction of BRD, AD, and PD activation were associated with the similarity of the activation profile of E Flexor, S Add/Flex, and S Abd/Ext, respectively, between age-matched control and stroke groups. In addition, we found that the preferred direction of these specific muscles’ activation after stroke, which increased the similarity of muscle synergies to the control, coincided with the preferred direction that led to improvements in motor impairment and qualities of isometric motor performance. These results indicate that rehabilitation strategies that aim to restore the preferred direction of individual muscle activation may enhance intermuscular coordination and qualities of motor performance and decrease motor impairments. Overall, the observations and reasoning may provide the scientific rationale to develop novel stroke neurorehabilitation strategies that emphasize the importance of improving the characteristics of individual muscle activation, such as the preferred direction of individual muscle activation.

## Data availability statement

The raw data supporting the conclusions of this article will be made available by the authors, without undue reservation.

## Ethics statement

The studies involving humans were approved by Northwestern University Institutional Review Board. The studies were conducted in accordance with the local legislation and institutional requirements. The participants provided their written informed consent to participate in this study.

## Author contributions

YH: Conceptualization, Methodology, Visualization, Writing – original draft, Writing – review and editing, Formal analysis. JR: Conceptualization, Data curation, Funding acquisition, Investigation, Project administration, Supervision, Writing – review and editing.

## Funding

The author(s) declare financial support was received for the research, authorship, and/or publication of this article. This study was supported by the American Heart Association Scientist Development Grant (17SDG33670561) and NSF CAREER Award (2145321).

## Conflict of interest

The authors declare that the research was conducted in the absence of any commercial or financial relationships that could be construed as a potential conflict of interest.

## Publisher’s note

All claims expressed in this article are solely those of the authors and do not necessarily represent those of their affiliated organizations, or those of the publisher, the editors and the reviewers. Any product that may be evaluated in this article, or claim that may be made by its manufacturer, is not guaranteed or endorsed by the publisher.
